# Causal link between prefrontal cortex and EEG microstates: evidence from patients with prefrontal lesion

**DOI:** 10.3389/fnins.2023.1306120

**Published:** 2023-12-14

**Authors:** Zongya Zhao, Xiangying Ran, Shiyang Lv, Junming Wang, Mengyue Qiu, Chang Wang, Yongtao Xu, Xiao Guo, Zhixian Gao, Junlin Mu, Yi Yu

**Affiliations:** ^1^School of Medical Engineering, Xinxiang Medical University, Xinxiang, China; ^2^Engineering Technology Research Center of Neurosense and Control of Henan Province, Xinxiang, China; ^3^Henan International Joint Laboratory of Neural Information Analysis and Drug Intelligent Design, Xinxiang, China; ^4^Henan Engineering Research Center of Medical VR Intelligent Sensing Feedback, Xinxiang, China; ^5^The First Affiliated Hospital of Xinxiang Medical University, Weihui, China; ^6^The Second Affiliated Hospital of Xinxiang Medical University, Xinxiang, China

**Keywords:** prefrontal lesion, causal link, EEG, microstate, prefrontal cortex

## Abstract

**Introduction:**

At present, elucidating the cortical origin of EEG microstates is a research hotspot in the field of EEG. Previous studies have suggested that the prefrontal cortex is closely related to EEG microstate C and D, but whether there is a causal link between the prefrontal cortex and microstate C or D remains unclear.

**Methods:**

In this study, pretrial EEG data were collected from ten patients with prefrontal lesions (mainly located in inferior and middle frontal gyrus) and fourteen matched healthy controls, and EEG microstate analysis was applied.

**Results:**

Our results showed that four classical EEG microstate topographies were obtained in both groups, but microstate C topography in patient group was obviously abnormal. Compared to healthy controls, the average coverage and occurrence of microstate C significantly reduced. In addition, the transition probability from microstate A to C and from microstate B to C in patient group was significantly lower than those of healthy controls.

**Discussion:**

The above results demonstrated that the damage of prefrontal cortex especially inferior and middle frontal gyrus could lead to abnormalities in the spatial distribution and temporal dynamics of microstate C not D, showing that there is a causal link between the inferior and middle frontal gyrus and the microstate C. The significance of our findings lies in providing new evidence for elucidating the cortical origin of microstate C.

## 1 Introduction

In recent years, EEG microstates analysis has become a popular method to characterize the neural activity of the whole brain in both space and time domain. EEG microstates that usually last for 60–120 ms are transient, spatially stable and recurrent patterns of brain activity visible in the EEG signal (Schiller et al., 2023). In general, there exists four typical EEG microstates (labeled as class A, B, C, and D) originally identified by Koenig et al. (1999), and these four microstates have always dominated the EEG data in different conditions although some studies have found more microstates (Gallotto and Seeck, 2022). The discovery of EEG microstates provides a new perspective for studying EEG signals and allows us to have a better understanding of the underlying mechanisms of brain function and information processing (Tarailis et al., 2023). Currently, EEG microstates have been widely applied in studying brain cognition (Jabès et al., 2021; Hua and Li, 2023), mental (de Bock et al., 2020; Mackintosh et al., 2020; Murphy et al., 2020) and neurological disorders (Chu et al., 2020; Tait et al., 2020; Toplutaş et al., 2023).

Although its wide application, a key scientific issue about EEG microstate that urgently needs to be addressed in the academic community is that these four typical microstates are associated with which brain regions. Several studies using concurrent EEG and fMRI technique have found significant neurophysiological correlations between the four EEG microstates and resting state networks (Yuan et al., 2012; Michel and Koenig, 2018). Specifically, microstate A is associated with the auditory, sensory, and somatomotor networks, which are primarily involved in bilateral temporo-parietal cortex and mesocortex (Britz et al., 2010). The visual network, which includes the bilateral occipital cortex, striatum, and extrastriate cortex, is linked to microstate B (Al Zoubi et al., 2022). The default mode network and executive control network mainly including the bilateral insula, bilateral inferior frontal cortex, and anterior cingulate cortex have close relationships with microstate C (Musso et al., 2010). The right frontal lobe, dorsoparietal lobe, and ventral cortex are all involved in the attention network, which is associated to microstate D (Bréchet et al., 2019). In addition to concurrent EEG and fMRI technique, several studies based on source localization analysis tried to explore the link between EEG microstates and brain regions. In a word, the superior temporal gyrus, medial prefrontal cortex and occipital brain gyrus are associated with microstate A; the medial parietal cortex and precuneus cortex are related to microstate B; microstate C has relationship with the lateral prefrontal cortex and the parietal cortex; and microstate D has connection with the bilateral inferior frontal gyrus, the dorsal anterior cingulate cortex and the parietal superior colliculus/medial parietal sulcus (Custo et al., 2017; Bréchet et al., 2019; Bagdasarov et al., 2022). The above-mentioned results suggest that microstates C and D have a close relationship with default mode network, executive control network and attention network, respectively, in which the prefrontal cortex plays an important role. Therefore, prefrontal lesions may affect the EEG microstate. But whether a causal link exists between prefrontal cortex and EEG microstate C or D is still unknown.

Based on the above researches, we hypothesize that there exists a causal link between prefrontal cortex (pfc) and EEG microstate C or D. At present, brain lesion is one of the most popular methods for studying the causal relationship between the brain area and behaviors or other factors, especially in animal research. In this study, in order to test our hypothesis, we obtained pretrial EEG data from patients with prefrontal lesions and carried out EEG microstate analysis to compare the differences of microstate between patients and healthy controls. Our expected result is that prefrontal damages can cause significant abnormality of microstate C or D. To the best of our knowledge, this is the first study to examine the causal relationship between prefrontal lobe especially inferior and middle frontal gyrus and microstate C or D.

## 2 Materials and methods

### 2.1 Subjects and EEG data

The EEG data used in present study were downloaded from Johnson et al. (2017), and the subjects, task and EEG recording have been described in detail in Johnson et al. (2017), Davoudi et al. (2021), and Parto Dezfouli et al. (2021). In brief, 14 adult patients with PFC lesions (pfc group) (mean ± SD: 46 ± 16 years of age, 15 ± 3 years of education, males/females: 5/9) and 20 healthy controls (ctrl group) (44 ± 19 years of age, 16 ± 3 years of education, males/females: 11/9) were recruited. Lesions are unilateral (*n* = 7 left hemisphere + 7 right hemisphere) and mainly focused in the inferior and middle frontal gyrus. Details of the lesions in patients with PFC lesions can be found in the Supplementary Information to this online article at https://doi.org/10.1016/j.cub.2017.05.046 (Johnson et al., 2017). All subjects have normal/corrected vision, an estimated IQ at least within the normal range, and no other neurological or psychiatric diagnoses. In our study, we screened the EEG data from this database to exclude subjects with poor EEG quality, and finally 10 patients [mean ± SD (range): 46.4 ± 16.5 (22–71) years of age, 14.2 ± 3.4 years of education, males/females: 4/6] and 14 matched healthy controls [38.8 ± 19.7 (19–70) years of age, 15.5 ± 2.5 years of education, males/females: 7/7] were left for further analysis, and their detailed information were listed in Table 1.

**TABLE 1:** Detailed information of subjects used in this study.

pfc group					ctrl group		
Subject name	Lesion hemisphere	Lesion etiology	Gender	Test age	Subject name	Gender	Test age
pfc 01	L	Astrocytoma grade II	F	48	ctrl 01	F	20
pfc 02	L	Cavernous hemangioma	M	54	ctrl 02	F	19
pfc 04	L	Stroke	M	34	ctrl 03	M	21
pfc 05	L	Stroke	F	64	ctrl 04	M	21
pfc 07	L	Stroke	F	71	ctrl 05	M	19
pfc 08	R	Ganglioglioma	F	22	ctrl 07	M	45
pfc 10	R	Cavernous hemangioma	M	41	ctrl 08	M	62
pfc 11	R	Cavernous hemangioma	M	37	ctrl 09	M	70
pfc 12	R	Astrocytoma grade II	F	29	ctrl 11	F	43
pfc 14	R	Stroke	F	64	ctrl 12	M	65
					ctrl 14	F	41
					ctrl 17	F	64
					ctrl 18	F	33
					ctrl 19	F	20

pfc group stands for patients with prefrontal lesion; ctrl group stands for healthy controls. L, stands for left hemisphere; R, stands for right hemisphere; F, stands for female; M, stands for male.

In the experimental paradigm involved in this database, 120–240 trials were performed per subject, and each trial was preceded by a 2-s pretrial, which is an open-eye fixation task. Therefore, each subject had at least 240 s of pretrial EEG data. However, after screening, we removed artifacts and noisier pretrials and retained a uniform of 60 pretrials (a total of 120 s) per subject for subsequent analysis.

EEG data were recorded using a 64+8 channel BioSemi ActiveTwo amplifier with Ag-AgCl pin-type active electrodes mounted on an elastic cap according to the International 10-10 System, sampled at 1024 Hz.

### 2.2 EEG data pre-processing

Offline EEG data preprocessing is performed by using MATLAB 2013b software (The MathWorks Inc. Natick, MA, USA) equipped with EEGLAB toolbox (Delorme and Makeig, 2004). Firstly, a zero-phase FIR band-pass filter is used to filter EEG data between 1 and 30 Hz (Strik et al., 1995). Secondly, the EEG data were down-sampled to 256 Hz. Thirdly, 2-s pretrial data epoch of each trial was extracted. Then, electrooculogram, electromyography, heartbeat and bad channels are identified and removed by using independent component analysis (Liang et al., 2021), and the average number of artifactual independent components was 2.21 ± 1.051 (mean ± std) and 2.2 ± 0.919 (mean ± std) for ctrl group and pfc group, respectively. Finally, the data were visually checked and epochs that still contain artifacts were removed, and all the EEG data were re-referenced to the common average reference. At last, sixty 2-s data epochs were selected from each subject and used for subsequent microstate analysis.

### 2.3 Microstates analysis

**FIGURE 1:**
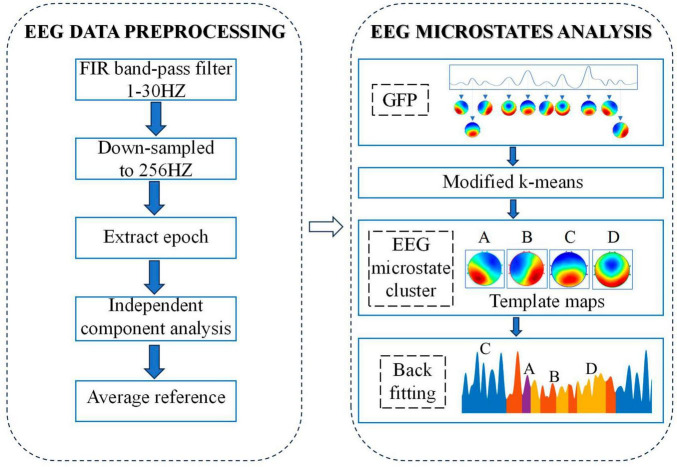
Flow chart of EEG data pre-processing and microstate analysis.

A microstate analysis toolbox based on EEGLAB toolbox was used to carried out microstate analysis (Poulsen et al., 2018). The process of analyzing EEG microstates is shown in Figure 1. In brief, the first step is to calculate the global field power (GFP) for each participant at each time point by using the following equation:


(1) $$\text{GFP}=\sqrt{\frac{\sum_{\text{i}=1}^{\text{n}}{\text{u}}_{\text{i}}^{2}}{\text{n}}}$$

where *i* denotes each electrode, *u* is EEG potential of each channel and *n* stands for the number of electrodes (here *n* = 64). Based on the above equation, we could get the GFP curve which reflects the overall potential variance across all electrodes. Because the EEG data around the local maximum of the GFP curve has the highest signal-to-noise ratio, topographic maps correspond to time point at the local maximum of GEP curve (called original maps) were chosen for the subsequent clustering analysis.

Based on the chosen original maps, a modified k-means was applied for clustering analysis while ignoring spatial polarity of maps. In order to determine the optimal number of clusters, a cross-validation criterion was applied while setting the number of clusters from 2 to 8, and the optimal number of clusters is 4. The original map of each subject was then clustered into four types of microstates. For the pfc group, by grouping and clustering the four microstates of all subjects in the group, we get the average original map of the pfc group, called the template map. In the same way, we can get the template map of the ctrl group. Finally, based on the spatial correlation between the template map and the scalp topographic map at each time point of the subjects in the corresponding group, the scalp topographic map at each time point of the subjects was divided into four types of microstates. After the above microstate analysis, the related EEG microstate temporal measures for each subject were extracted:

(a) Duration (Mean microstate duration, Unit:ms): the average duration for each microstate.
(b) Coverage (Ratio of total time covered): time coverage percentage of each microstate class in total analysis time.
(c) Occurrence (Times per second): The average number of occurrences for each microstate in 1 s.
(d) Transition probability: the transition percentage from each of the four microstate classes to any other class. For example, the transition probability from microstate A to microstate B, is defined as the percentage of the transfer times from A to B over the total transfer times from A to other three microstates (Liu et al., 2023).

It is noted that the transition probability and coverage are in percentage, and the units of duration and occurrence are millisecond and times per second, respectively.

### 2.4 Statistical analysis

SPSS 19.0 software was used for the statistical analysis, and data were represented as mean ± standard deviation. The Shapiro–Wilk test was used to test for normality of distribution. The independent sample *t*-test was used to assess group differences of the microstate parameters (duration, occurrence, coverage, transition probability), and false discovery rate (FDR) correction was applied to control the multiple comparison issue.

The statistical comparison of EEG topography between two or more groups/conditions can be done using the Topographic Analysis of Variance (TANOVA), which is based on robust and assumption-free randomization statistics. In this study, TANOVA analysis was carried out using a toolbox named Ragu (Koenig et al., 2002, 2011) to explore whether there exists a statistical difference of microstate A/B/C/D between patient group and healthy controls.

## 3 Results

### 3.1 EEG microstate topographies

**FIGURE 2:**
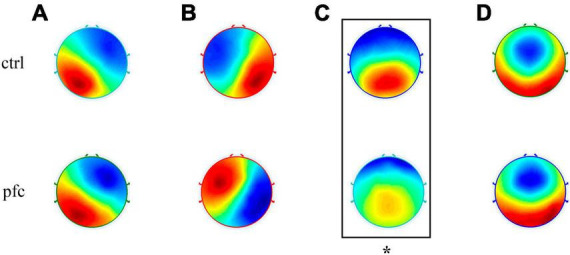
Topographic maps of four types of EEG microstates (microstates **A–D**) in ctrl and pfc group. The asterisk showed that there existed a significant difference for the corresponding microstate topography between two groups (*P* < 0.05).

Microstate analysis based on clustering algorithm was used to analyze the topographic maps of pfc and ctrl group, and the EEG topographies of each group were classified into four types of microstates according to cross-validation criterion. As shown in Figure 2, our obtained four microstates were consistent with the classical four microstates (named A, B, C and D, respectively) reported in most previous studies (Koenig et al., 1999, 2002; Michel and Koenig, 2018; Pan et al., 2021; Tamburro et al., 2021). In brief, microstate class A has a left occipital to right frontal orientation which exhibits a left-right orientation, class B was from right occipital to left frontal that exhibits a right-left orientation, class C has a symmetric occipital to prefrontal orientation which is an anterior-posterior orientation and class D was also symmetric, but with a frontocentral to occipital axis which exhibits a fronto-central maximum. However, the spatial distribution of microstate C in pfc group seems to be different from that of ctrl group visually. In order to explore whether there exists significant difference of microstate C topography between the two groups, the TANOVA method was applied and results showed that there was significant difference in the topographic map of microstate C between the two groups (*P* = 0.0226 < 0.05). For microstate A, B, and D, no significant differences in the topographic maps between the two groups were found (*P* > 0.05). At the same time, to assess the extent to which the original EEG data was interpreted by the microstate topographic maps, we statistically analyzed the global explained variance (GEV) between the two groups. Results showed that the GEV of ctrl and pfc group was 0.6546 ± 0.0633 and 0.6035 ± 0.0536, respectively, and independent sample *t*-test showed that there was no statistical difference in GEV between the two groups (*t* = 1.7388, *p* = 0.0960 > 0.05).

### 3.2 EEG microstate parameters

**FIGURE 3:**
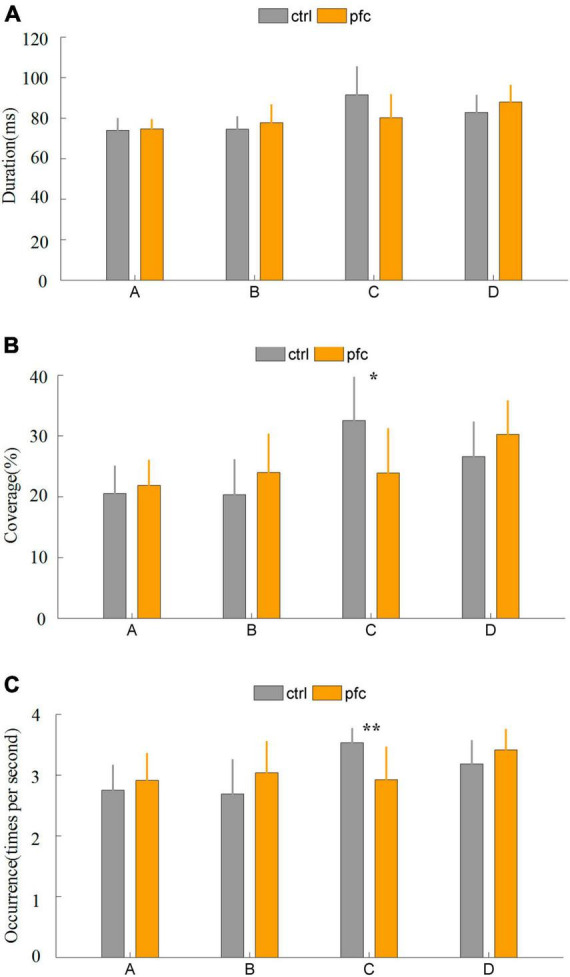
Comparison of three microstate temporal parameters between pfc and ctrl group: **(A)** duration, **(B)** coverage, **(C)** occurrence. “**” and “*” stands for *P*_fdr_ < 0.01 and *P*_fdr_ < 0.05, respectively.

Some important temporal parameters that can characterize brain network dynamics can be obtained from microstate analysis. In this study, duration, coverage, occurrence and transition probability were extracted to quantitatively explore the differences between pfc and ctrl group. It was found that the duration of the four microstates did not differ significantly between the two groups (Figure 3A). As for coverage, pfc group showed significantly lower mean coverage of microstate classes C compared to ctrl group (Figure 3B and Table 2; *t* = 2.8692, *P* = 0.0089, *P*_*fdr*_ = 0.0357 < 0.5). As show in Figure 3C and Table 2, pfc group also showed significantly lower mean occurrence of microstate classes C compared to ctrl group (*t* = 3.6961, *P* = 0.0013, *P*_fdr_ = 0.0050 < 0.5).

**TABLE 2:** Microstate temporal parameters with statistical differences.

	ctrl group		pfc group				
	Mean	S.D.	Mean	S.D.	*t*-value	*P*	*P*_fdr_
**Coverage (%)**							
Class C	32.54	7.19	23.91	7.37	2.8692	0.0089	0.0357
**Occurrence**							
Class C	3.5313	0.2442	2.9249	0.5456	3.6961	0.0013	0.0050
**Transition probability (%)**							
A→C	38.79	5.76	29.10	6.70	3.6332	0.0015	0.0176
B→C	39.89	5.83	31.43	6.44	3.2127	0.0040	0.0241

At the same time, the transition probability between the pfc group and the ctrl group is also compared. The results showed that pfc group exhibited significantly lower transition probability from microstates A to C (*t* = 3.6332, *p* = 0.0015, *P*_fdr_ = 0.0176 < 0.05) and from B to C (*t* = 3.2127, *p* = 0.0040, *P*_fdr_ = 0.0241 < 0.05) compared with ctrl group (Table 2). The mean and standard deviation of all microstate parameters are shown in Supplementary Table 1.

## 4 Discussion

In this study, we used EEG microstate analysis to study whether prefrontal lesion could lead to abnormal changes of microstates. We found that the EEG microstate C topography was significantly abnormal in pfc group compared to ctrl group. In addition, compared to ctrl group, coverage and occurrence of microstate C, and average transition frequency of microstate A to C and B to C were significantly decreased in pfc group. Our findings provide new evidence for elucidating a causal link between the prefrontal cortex especially inferior and middle frontal gyrus and the microstate C.

The prefrontal lobe is an important region of the cerebral cortex that is thought to be central to working memory, goal-driven attention, task switching, planning, problem-solving and novelty-seeking (MacDonald et al., 2000; Baddeley, 2003; Kesler et al., 2011). And the lesion areas in pfc group were mainly located in inferior and middle frontal gyrus. In this study, we found that EEG microstate C topography was significantly abnormal in patients with prefrontal lesion with a symmetrical occipital-to-prefrontal and anterior-posterior orientation distribution diminishing compared to ctrl group. Based on concurrent EEG and fMRI technique, Al Zoubi et al. (2022) found that microstate C has a significant relationship with left middle frontal gyrus. Britz et al. (2010) proved that microstate C is correlated with fMRI activity in bilateral inferior frontal gyri. Custo et al. (2017) used EEG source localization analysis to study the cortical origins of EEG microstates and also found that microstate C was closely related to bilateral middle frontal gyrus. A study of the spatiotemporal dynamics of EEG microstates in children also showed that microstate C was associated with right inferior frontal gyrus and bilateral middle, medial and superior frontal gyri (Bagdasarov et al., 2022). These studies actually support our findings to some extent, although they do not provide causal evidence. However, Britz et al. (2010) and Custo et al. (2017) also found that microstate D is related to middle frontal gyrus, which is not consistent with our findings, because our results did not find that the damage of inferior and middle frontal gyrus could lead to abnormal changes of microstate D. Therefore, from our findings, we believed that the inferior and middle frontal gyrus are major cortical origins of microstate C not D.

In addition to abnormal spatial distribution of microstate C topography, we found that the mean coverage and occurrence of microstate C significantly decreased compared to ctrl group. Based on the previous finding that microstate C was closely related to executive control network (ECN) (Croce et al., 2018), our results might indicate that ECN showed a state of severe inactivation and inhibition in pfc group, which might explain why pfc group exhibited very poor performance in working memory task (Johnson et al., 2017).

In recent years, some non-invasive neuromodulation techniques such as repetitive transcranial magnetic stimulation (rTMS) and transcranial direct current stimulation (tDCS) have become popular methods for treating mental and neurological disorders. And these techniques also provide the possibility for studying the causal relationship between the brain area and behaviors or other factors, especially in human researches. A pilot study that using rTMS to stimulate the dorsolateral prefrontal cortex of schizophrenic patients found that the occurrence of only microstate C in patients who respond to treatment significantly decreased after stimulation (Sverak et al., 2018). A similar study carried out by Pan et al. (2021) also found that schizophrenic patients receiving rTMS showed a decreasing trend in the prevalence of microstate C. Chen et al. (2022) applied anodal tDCS on the left dorsolateral prefrontal cortex for treating disorders of consciousness and found a significant increase in microstate C coverage in patients who responded to stimulation. Although these studies involved mental and neurological patients, they provide evidences to some extent to prove the causal relationship between the prefrontal lobe and microstate C, which also confirmed our finding in this study.

In addition, we noted a significant reduction in the average transition frequency of microstate A to C and B to C in the pfc group. Considering that microstate C is associated with the default mode network and executive control network (Musso et al., 2010), and microstates A and B are associated with the sensorimotor and visual networks, respectively (Britz et al., 2010; Al Zoubi et al., 2022), we speculated that the lower transition from microstates A and B to microstate C in pfc patients may reflect abnormalities in network-to-network functional connectivity and transmission, which needs further validation in future researches.

At present, the research trend of task-state electroencephalography is increasing, especially the research of electroencephalography involving human emotions. Human emotion is one of the cognitive functions of the brain, which is mainly related to the prefrontal cortex. Wright et al. (2004) believed that disgust emotion is related to the activation of the insula and the ventral prefrontal cortex. A recent study examined the microstate characteristics of nine emotions (anger, disgust, fear, sadness, neutrality, amusement, inspiration, joy, and tenderness) (Liu et al., 2023). Based on the characteristics of brain microstate under neutral emotions, the study found that the coverage of microstate C could be regarded as the most important feature, and the coverage of microstate C under both positive and negative emotions was higher than that under neutral emotions. In the future, it will be an interesting research direction to explore the emotion-related characteristics of EEG microstate in patients with prefrontal lesions.

However, there are still some limitations that should be considered in the future. Firstly, the number of patients with prefrontal lesions included in this study is relatively small, and more patients are needed to confirm our results. Secondly, although the lesion areas of the patients were mainly located in the inferior and middle frontal gyrus, the size and location of each patient’s lesion are not entirely consistent, which may have some impact on the results. Finally, whether the pretrial EEG data in this paper is essentially consistent with the resting EEG data needs to be further verified. In addition, non-invasive techniques such as rTMS and tDCS can be used to further explore the causal relationship between prefrontal cortex and EEG microstate C.

## 5 Conclusion

This study showed that the damage of prefrontal cortex especially inferior and middle frontal gyrus could lead to abnormalities in the spatial distribution and temporal dynamics of microstate C instead of microsite A, B, and D, showing that there is a causal link between the inferior and middle frontal gyrus and the microstate C. In brief, the current study provides new evidence for elucidating the cortical origin of microstate C.

## Data availability statement

The original contributions presented in the study are included in the article/Supplementary material, further inquiries can be directed to the corresponding authors.

## Ethics statement

All subjects gave informed written consent in accordance with the University of California, Berkeley, Institutional Review Board or the Regional Committee for Medical Research Ethics, Region South, and in agreement with the Declaration of Helsinki. The studies were conducted in accordance with the local legislation and institutional requirements. The participants provided their written informed consent to participate in this study. Written informed consent was obtained from the individual(s) for the publication of any potentially identifiable images or data included in this article.

## Author contributions

ZZ: Data curation, Funding acquisition, Investigation, Methodology, Writing – review and editing, XR: Investigation, Writing – original draft. SL: Writing – original draft. JW: Writing – original draft. MQ: Writing – original draft. CW: Writing – review and editing. YX: Writing – review and editing. XG: Writing – review and editing. ZG: Writing – review and editing. JM: Writing – review and editing. YY: Writing – review and editing.
