# Supplementary material for: Causal link between prefrontal cortex and EEG microstates: evidence from patients with prefrontal lesion
Source: Front Neurosci. 2023 Dec 14;17:1306120. doi: 10.3389/fnins.2023.1306120 (PMC10757643; doi:10.3389/fnins.2023.1306120)
Supplement: Supplementary file 1 [file Table_1.docx]

Table S1. the mean and standard deviation of all microstate parameters

|  | ctrl group | |  | pfc group | |  |  |  |  |  |
| --- | --- | --- | --- | --- | --- | --- | --- | --- | --- | --- |
|  | Mean | S.D. |  | Mean | S.D. |  | t value |  | P | P_fdr_ |
| **Coverage(%)** |  |  |  |  |  |  |  |  |  |  |
| Class A | 20.53 | 4.60 |  | 21.87 | 4.18 |  | -0.7876 |  | 0.4722 | 0.4722 |
| Class B | 20.34 | 5.83 |  | 23.98 | 6.41 |  | -1.1341 |  | 0.1611 | 0.2148 |
| Class C | 32.54 | 7.19 |  | 23.91 | 7.37 |  | 2.8692 |  | 0.0089 | 0.0357 |
| Class D | 26.60 | 5.78 |  | 30.24 | 5.62 |  | -1.4340 |  | 0.1379 | 0.2148 |
| **Duration(ms)** |  |  |  |  |  |  |  |  |  |  |
| Class A | 73.9609 | 6.1298 |  | 74.7001 | 4.7840 |  | -0.3637 |  | 0.7537 | 0.7537 |
| Class B | 74.4835 | 6.3985 |  | 77.7630 | 8.9433 |  | -0.8411 |  | 0.3051 | 0.4069 |
| Class C | 91.4594 | 14.1093 |  | 80.1697 | 11.6983 |  | 1.8341 |  | 0.0505 | 0.2018 |
| Class D | 82.7128 | 8.7648 |  | 87.9423 | 8.4906 |  | -1.4164 |  | 0.1585 | 0.3171 |
| **Occurrence (Ratio of total time covered)** |  |  |  |  |  |  |  |  |  |  |
| Class A | 2.7494 | 0.4209 |  | 2.9122 | 0.4503 |  | -0.9494 |  | 0.3737 | 0.3737 |
| Class B | 2.6899 | 0.5711 |  | 3.0392 | 0.5208 |  | -1.2436 |  | 0.1400 | 0.1952 |
| Class C | 3.5313 | 0.2442 |  | 2.9249 | 0.5456 |  | 3.6961 |  | 0.0013 | 0.0050 |
| Class D | 3.1812 | 0.3940 |  | 3.4144 | 0.3435 |  | -1.3698 |  | 0.1464 | 0.1952 |
| **Transition probability(%)** |  |  |  |  |  |  |  |  |  |  |
| A→B | 27.34 | 6.86 |  | 32.56 | 7.15 |  | 1.1280 |  | 0.0983 | 0.1928 |
| A→C | 38.79 | 5.76 |  | 29.10 | 6.70 |  | 3.6332 |  | 0.0015 | 0.0176 |
| A→D | 33.86 | 6.11 |  | 38.34 | 6.47 |  | 1.4560 |  | 0.1125 | 0.0928 |
| B→A | 27.27 | 5.34 |  | 30.42 | 6.91 |  | 1.3770 |  | 0.2403 | 0.3240 |
| B→C | 39.89 | 5.83 |  | 31.43 | 6.44 |  | 3.2127 |  | 0.0040 | 0.0241 |
| B→D | 32.84 | 4.65 |  | 38.15 | 5.41 |  | 1.9390 |  | 0.0220 | 0.0528 |
| C→A | 30.48 | 4.87 |  | 29.30 | 4.09 |  | -2.9960 |  | 0.5560 | 0.6672 |
| C→B | 30.77 | 7.57 |  | 31.83 | 6.71 |  | -2.7710 |  | 0.7388 | 0.8059 |
| C→D | 38.75 | 7.57 |  | 38.87 | 7.64 |  | -2.1670 |  | 0.9703 | 0.9703 |
| D→A | 28.97 | 5.67 |  | 32.38 | 5.73 |  | 1.6630 |  | 0.1796 | 0.2694 |
| D→B | 26.81 | 6.46 |  | 33.74 | 5.77 |  | 2.0250 |  | 0.0167 | 0.0528 |
| D→C | 44.22 | 9.27 |  | 33.89 | 9.48 |  | 0.0010 |  | 0.0181 | 0.0528 |
